# Comment on “Efficacy and Safety of Topical Application of Plant‐Based Products on Skin Aging in Healthy Individuals: A Systematic Review and Meta‐Analysis of Randomized Controlled Trials”

**DOI:** 10.1111/jocd.70110

**Published:** 2025-03-10

**Authors:** Mehrab Neyazi, Rachana Mehta, Shubham Kumar, Sanjit Sah

**Affiliations:** ^1^ Afghanistan Center for Epidemiological Studies Herat Afghanistan; ^2^ Faculty of Medicine Ghalib University Herat Afghanistan; ^3^ Clinical Microbiology, RDC Manav Rachna International Institute of Research and Studies Faridabad Haryana India; ^4^ Center for Global Health Research Saveetha Medical College and Hospital, Saveetha Institute of Medical and Technical Sciences, Saveetha University Chennai India; ^5^ SR Sanjeevani Hospital Kalyanpur Siraha Nepal; ^6^ Department of Paediatrics Dr. D. Y. Patil Medical College Hospital and Research Centre, Dr. D. Y. Patil Vidyapeeth (Deemed‐To‐Be‐University) Pune Maharashtra India; ^7^ Department of Public Health Dentistry Dr. D. Y. Patil Medical College Hospital and Research Centre, Dr. D. Y. Patil Vidyapeeth (Deemed‐To‐Be‐University) Pune Maharashtra India

**Keywords:** age, plants, wrinkles

Dear Editor,

We read with great interest the study by Cheng et al. [1], titled “Efficacy and Safety of Topical Application of Plant‐Based Products on Skin Aging in Healthy Individuals: A Systematic Review and Meta‐Analysis of Randomized Controlled Trials”. The authors provide a comprehensive evaluation of the effects of plant‐based products on various markers of skin aging, offering a valuable contribution to this growing field of dermatological research. The findings, particularly the demonstrated improvements in skin hydration, elasticity, and reductions in melanin and erythema, provide a promising basis for future research and clinical applications. However, we believe there are a few areas where methodological refinements could enhance the robustness and clinical applicability of this review.

While the study utilized the Cochrane Risk of Bias (ROB) tool to assess the quality of the included randomized controlled trials, performing a sensitivity analysis based on study quality specific to skin aging outcomes would have been valuable [2]. For example, analyzing how the exclusion of studies with a high risk of bias affected pooled results for markers such as skin hydration, elasticity, or melanin reduction could provide critical insights into the robustness of the reported effects. This approach would enhance confidence in the conclusions by isolating the influence of potential biases on the observed benefits of plant‐based products.

A Grading of Recommendations, Assessment, Development, and Evaluations (GRADE) assessment was not performed, which could have provided a systematic evaluation of the certainty of evidence for each skin‐aging outcome [3, 4]. Incorporating GRADE would allow for clearer distinctions between the strength of evidence supporting findings such as improvements in hydration and elasticity versus less conclusive outcomes like transepidermal water loss (TEWL). Additionally, this would aid in developing clinical recommendations by addressing evidence quality factors such as imprecision in measurements, variability across studies, and relevance to broader populations, ultimately enhancing the practical application of these findings in dermatological practice.

We suggest that future research could benefit from exploring the specific mechanisms of action of plant‐based products in mitigating skin aging. While the meta‐analysis shows improvements in markers such as hydration and elasticity, it does not delve into the molecular or cellular pathways affected by these products. Identifying the active components (e.g., phenolics, terpenes) and their specific roles in combating oxidative stress, inflammation, and other aging processes could bridge the gap between clinical outcomes and mechanistic understanding.

This study represents a significant advancement in the exploration of plant‐based products for skin aging. By incorporating sensitivity analyses, GRADE assessments, and mechanistic investigations, future studies can further refine and substantiate these findings. We appreciate the authors' valuable contributions to this field and look forward to seeing how this research evolves.

## Author Contributions

R.M., S.S., M.N. critically provided comments on methodological aspects. M.N., S.K. wrote and edited the draft.

## Ethics Statement

The authors have nothing to report.

## Consent

The authors have nothing to report.

## Conflicts of Interest

The authors declare no conflicts of interest.

## Data Availability

The authors have nothing to report.
